# High-intensity focused ultrasound ablation around the tubing

**DOI:** 10.1371/journal.pone.0188206

**Published:** 2017-11-21

**Authors:** Jun Yang Siu, Chenhui Liu, Yufeng Zhou

**Affiliations:** School of Mechanical and Aerospace Engineering, Nanyang Technological University, Singapore, Singapore; Nanjing University, CHINA

## Abstract

High-intensity focused ultrasound (HIFU) has been emerging as an effective and noninvasive modality in cancer treatment with very promising clinical results. However, a small vessel in the focal region could be ruptured, which is an important concern for the safety of HIFU ablation. In this study, lesion formation in the polyacrylamide gel phantom embedded with different tubing (inner diameters of 0.76 mm and 3 mm) at varied flow speeds (17–339 cm/s) by HIFU ablation was photographically recorded. Produced lesions have decreased length (~30%) but slightly increased width (~6%) in comparison to that without the embedded tubing. Meanwhile, bubble activities during the exposures were measured by passive cavitation detection (PCD) at the varied pulse repetition frequency (PRF, 10–30 Hz) and duty cycle (DC, 10%-20%) of the HIFU bursts. High DC and low flow speed were found to produce stronger bubble cavitation whereas no significant influence of the PRF. In addition, high-speed photography illustrated that the rupture of tubing was produced consistently after the first HIFU burst within 20 ms and then multiple bubbles would penetrate into the intraluminal space of tubing through the rupture site by the acoustic radiation force. Alignment of HIFU focus to the anterior surface, middle, and posterior surface of tubing led to different characteristics of vessel rupture and bubble introduction. In summary, HIFU-induced vessel rupture is possible as shown in this phantom study; produced lesion sizes and shapes are dependent on the focus alignment to the tubing, flow speed, and tubing properties; and bubble cavitation and the formation liquid jet may be one of the major mechanisms of tubing rupture as shown in the high-speed photography.

## Introduction

High-intensity focused ultrasound (HIFU) has become more popular in clinics in the treatment of cancers, such as uteroid fibroids and breast, liver, pancreas, prostate, kidney, prostate, and bone cancers, with very promising results since the middle of the 1990s [1]. Its advantages of noninvasiveness, a possible procedure in a doctor’s office or outpatient clinic without the use of a sterile operating room, few complications, less in-hospital cost and risk of metastasis in comparison to the open surgery, radio- and chemo-therapy. Most of the cancer and solid tumors are hypervascular so that whether HIFU ablation is hazardous to blood vessels in or close to the focal region is a critical concern for the safety of this novel technology.

In a 7 year-investigation of 39 patients with 42 hepatocellular carcinoma in the size of 7.4±4.3 (1.5–22) cm treated by HIFU with the distance between tumor and main blood vessel less than 1 cm, the perfusion of tumors and major blood vessels, the inferior vena cava, main hepatic vein branches, and the portal vein and its main branches were evaluated by contrast-enhanced MRI [2]. Twenty-one tumors were completely ablated, while more than 50% of lesion volumes in the rest after one HIFU session. No major blood vessel injury was observed in any subjects after 23.8±17.2 mo follow-up. However, in another investigation of 164 patients, enhanced MRI, color Doppler ultrasound imaging, dynamic radionuclide scanning, digital subtraction angiography, and histologic study were performed to monitor the response of tumor vessels [3]. An abrupt blood interruption was found followed by the cessation of blood flow within the tumor vessels after HIFU treatment. The histologic examination indicated not only coagulative necrosis but also severely damaged small tumor vessels. Furthermore, HIFU (e.g., a frequency of 1.51 MHz, a pulse duration of 0.1–10 ms, and acoustic powers up to 300 W) can also restore the blood flow in the thrombus occluded femoral arteries of rabbits *in vivo* only in the presence of cavitation without thrombolytic agents [4]. But HIFU exposure at the pulse duration of 10 ms was associated with the bleeding. In the meta-analysis of HIFU-induced vessel occlusion for treating the neoplastic pathology, the rupture was found to occur in 13% to 80% of the vessels, and this rate increased with the acoustic intensity [5]. However, tumor hemorrhage or rupture of large blood vessels has never been detected after HIFU treatment, which is thought to be because of the heat transfer by the blood flow or its great robustness to damage [6].

The mechanisms of HIFU-induced vessel rupture are not fully understood. One possible mechanism is supratherapeutic heating of the constricted vessel wall because the HIFU-generated heat was not dissipated due to the reduced convection [7]. Local tissue boiling by heating to 100°C, even at short pulse duration, with the acoustic radiation force plays a concomitant role in the mechanical erosion of tissue [8]. Inertial cavitation in the HIFU field, the violent collapse of microbubbles or high-velocity microjets, results in the substantially mechanical damage to the tissue [9–11]. Meanwhile, stable cavitation or the sustained large-amplitude oscillation of microbubbles is also believed to cause the mechanical effects due to microstreaming [12].

In this study, lesion formation during HIFU ablation with the focus aligned to the middle of the vessel phantom (different tubing with various materials, geometries, and flow velocities) was monitored in a polyacrylamide gel phantom by a digital camera and then quantified. Lesion sizes were compared with those in the gel phantom without embedded tubing. Passive cavitation detection (PCD) was performed to capture the bubble activities. Tubing rupture was consistently observed during the experiment, and multiple bubbles were pushed into the tubing from the HIFU-induced lesion through the rupture site. High-speed photography (1,000 frames/s) illustrated such a process clearly. With the HIFU focus aligned to the anterior and posterior surface of the tubing, different characteristics of tubing rupture, bubbes introduction, and lesion formation in the gel phantom were found. In summary, HIFU ablation to the tubing with its orientation aligned perpendicular to the HIFU axis can generate the lesion successfully as well as the tubing rupture. The size and shape of the lesion depend on the tubing material, geometry, and flow speed. The rupture of tubing was consistent by HIFU exposure (the peak negative pressure of -11.9 MPa in the degassed water) within the first 20 ms. Focus alignment to the tubing is also of importance for the induced damage. Bubble cavitation and produced jet may be one of the dominant mechanisms of tubing rupture.

## Materials and methods

### Experimental setup

The optically transparent gel phantom (L×W×H = 6×4.3×3.9 cm) was composed of polyacrylamide hydrogel and bovine serum albumin (BSA) and became optically opaque when thermally denatured at the temperature over 65°C [13]. The mixture of 40% w/v acrylamide solution (ICN Biomedicals, Aurora, OH) with a 19:1 ratio of acrylamide:bis and 10% w/v ammonium persulfate solution (Sigma-Aldrich, Singapore) and 7% BSA (Axil Scientific, Singapore) by weight was degassed for about 1 h in a desiccant chamber (420100000, Scienceware, Pequannock, NJ) with a vacuum pump (VTE8, Thomas, Sheboygan, WI) at a pressure of 150 mbars to remove the bubbles in the mixture, then poured into a mold, which has an acoustically transparent membrane at the bottom, for polymerization at room temperature within 1 min of adding N,N,N’,N’-tetra-methylethylene/diamine (TEMED, Sigma-Aldrich).

Tubing with different materials and dimensions (190-024-001, Cole-Parmer, Vernon Hills, IL and 1009, Polymed, New Delhi, India) was used as the vessel phantom and listed in Table 1. The diameter and wall thickness of small, medium, and large artery are 1–2.5 mm and 0.5 mm, 2.5–4 mm and 0.75 mm, 4–5 mm and 1 mm, respectively [14]. Two straight tube connectors (Cole-Parmer) were fixed on the mold, and tubing was routed between the connector barbs. The gap between the connectors and tubing was filled with the epoxy to prevent the liquid leakage. In order to minimize the bubble adhere to the tubing wall ethanol (Sigma-Aldrich) was smeared on the outer surface to decrease the surface tension before pouring the BSA gel solution. After the solidification of polyacrylamide hydrogel, the tubing was embedded inside the gel phantom. The connectors were inserted into large silicone tubing (EW-96101-18, Cole-Parmer) which was connected to a peristaltic pump (pump head: YZ1515x, BT300-2J, Longer Precision Pump, Baoding, China). Degassed and deionized water flowed from the reservoir to a fluid collector through the tubing.

**Table 1:** Geometries and properties of tubings used in the HIFU ablation.

	Tubing 1	Tubing 2
**inner diameter**	0.76 mm or 1/32”	3.0 mm
**outer diameter**	2.27 mm or 3/32”	4.1 mm
**material**	silicone rubber	PVC soft
**Young’s modulus (MPa)**	1–50	20–50
**specific heat capacity (J/kg/K)**	1050–1300	1400

**Fig 1:**
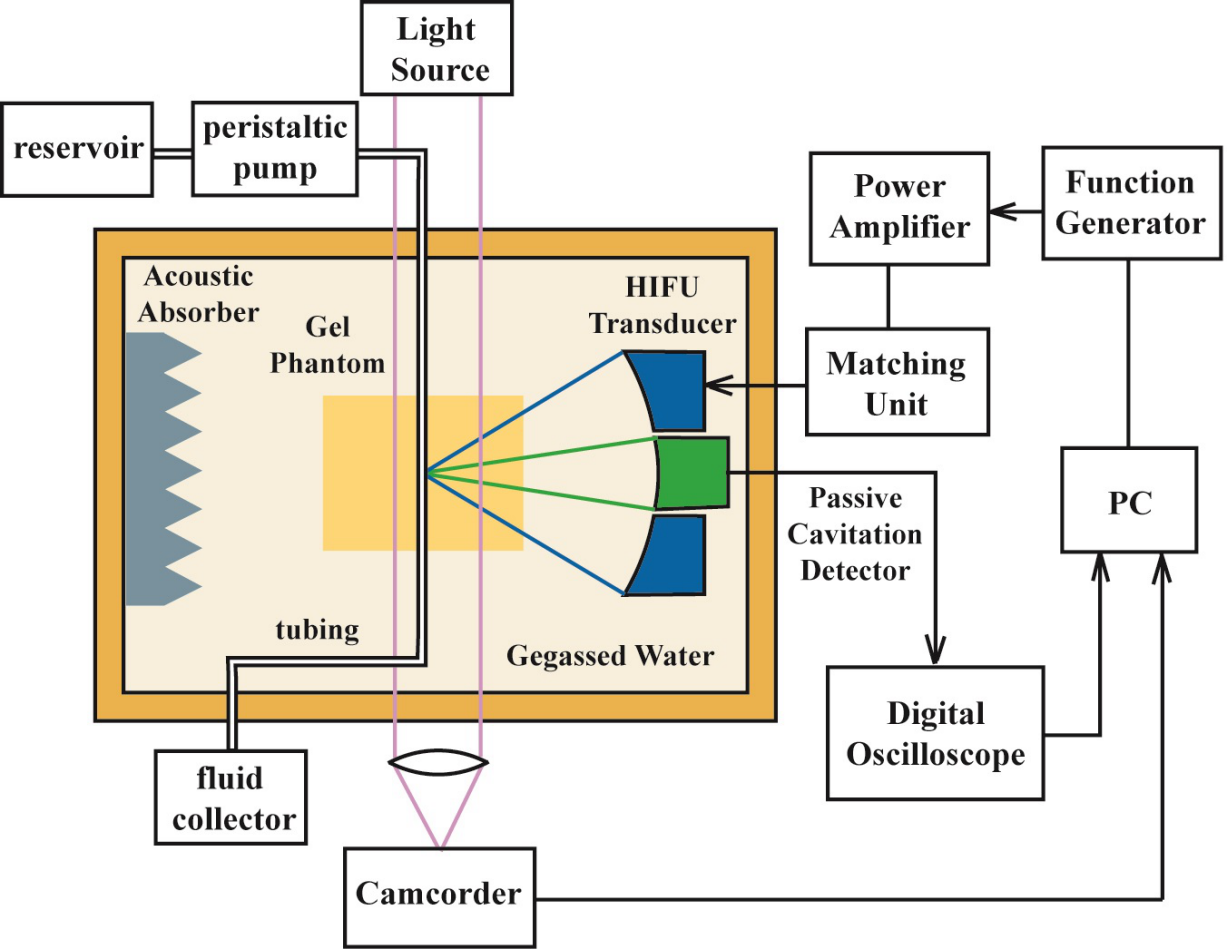
Schematic diagram of experimental setup for HIFU ablation for a tubing embedded in the gel phantom and passive cavitation detection.

A single-element HIFU transducer (H-102, outer diameter = 69.94 mm, inner diameter = 22.0 mm, *F* = 62.64 mm, Sonic Concepts, Woodinville, WA) working at its third harmonic frequency (3.3 MHz) was used in this study (Fig 1). The HIFU transducer was immersed in the degassed and deionized water (*O*_*2*_ < 4 mg/L, *T* = 25°C, measured by DO700, Extech Instrument, Waltham, MA) of a Lucite tank (L×W×H = 70×50×30 cm) and driven by sinusoidal bursts produced by a function generator (AF3021B, Tektronix, Beaverton, OR) together with a power amplifier (BT00250-AlphaA, Tomco Technologies, Adelaide, Australia). An acoustic absorber was placed on the opposite wall of the testing tank to prevent the ultrasound reflection. The HIFU transducer was attached to a three-axis positioning system (PT3/M, Thorlabs, Newton, NJ) to align its focus with respect to the tubing. The alignment to the anterior surface of tubing (outer membrane) was achieved by obtaining the maximum echo signal using a pulser/receiver (5072PR, Olympus-IMS, Waltham, MA), and then HIFU focus could be moved to the middle or posterior surface of the tubing by translating the transducer axially in a certain distance (radius or diameter of tubing) using the translational stage. A LabView program (National Instruments, Austin, TX) was written to set the HIFU parameters and control the pulse delivery (100 s). Any bubbles inside the tubing were flushed away by the circulating water before HIFU exposure, and the HIFU focus was moved transversely by at least 7 mm to minimize the influence from the previously treated region.

### Photography

A digital camera (PowerShot SX230 HS, Canon, Tokyo, Japan) was amounted on the top of the gel phantom holder to graphically record the lesion generation. After HIFU ablation, the captured images were processed in digital image processing software (Photoshop, Adobe System, San Jose, CA) to quantitatively determine the lesion size. A ruler was attached to the wall of the phantom holder for the calibration purpose.

The lesion formation in the BSA gel phantom, the rupture of tubing, and the dynamics of bubble inside the tubing were recorded using a high-speed camera (S-PRI plus, AOS Technologies AG, Dättwil, Switzerland), working at 1,000 frames per second in a resolution of 640 by 480 pixels. An optical lens (focal length from 12.5 to 53 mm, *f/1*.*8D*, Nikon, Tokyo, Japan) and a set of the close-up lens (Nikon) were mounted on the camera to achieve an appropriately magnified view. The camera was triggered by a transistor-transistor logic (TTL) pulse from a digital delay generator (DG535, Stanford Research Systems, Sunnyvale, CA) to synchronize the photography with HIFU exposure, and the recording started 10 ms before the delivery of HIFU pulses. Because of the limited memory in the camera, only one second of HIFU exposure was recorded. A 1000 W white light (Model H10, Hi-Tech Electronics, Singapore) was used as the illumination in the photography. The individual frame could be extracted from the captured videos using software (AOS imaging Studio V3, AOS Technologies AG) companied with the high-speed camera.

### Passive cavitation detection

A focused ultrasound probe (A319S, *f* = 15 MHz, *D* = 12.7 mm, *F* = 65 mm, Olympus-IMS, Waltham, MA) was aligned confocally and coaxially with the HIFU transducer and worked as a PCD sensor [15]. PCD signals of each HIFU burst were recorded by a digital oscilloscope (Wavesurfer MXs-B, LeCroy, Chestnut Ridge, NY) at a sampling frequency of 50 MHz and then transferred to a personal computer for data analysis in Matlab (The Mathworks, Natick, MA). Sequence mode was used in the data acquisition. Because of the limited memory of digital oscilloscope, the interval time was set as 10 s. Peak-to-peak and root mean square (rms) of PCD signals in each HIFU burst were used to quantify the cavitation levels.

### Statistical analysis

Analysis of variance (ANOVA) test was performed to determine the statistical difference between the groups that was fixed at *p* < 0.05 in SigmaPlot (Systat Software, San Jose, CA). The sample size at each testing condition was at least five.

## Results

The volume flow rates produced by the pulsatile pump at different rotation rates were measured by filling water into a 200 ml beaker, and then the average flow speed in the tubing was determined by dividing the volume flow by the inner sectional area of tubing and listed in Table 2. The average flow speed in the tubing 2 is within the range of reported blood flow in the human body (e.g., the aorta of 40 cm/s, vena cavae inferior and superior of 15 cm/s, the capillary of 0.03 cm/s) [14] while that in the tubing 1 is higher. The specific heat capacity of an artery is 3470 J/kg/K [16]. The HIFU transducer has -6 dB beam size of 4×0.5 mm (axial×lateral) as measured by a needle hydrophone (HNA-0400, Onda, Sunnyvale, CA) using the established scanning protocol [17], and the peak positive and negative pressures at the focus in the degassed water are about 34.2 and -11.9 MPa, respectively.

**Table 2:** Flow rate in the tubing.

pump speed (rpm)	volume flow rate (ml/min)	average flow speed (cm/s)	
		tubing 1	tubing 2
40	72.6	266.7	17.1
45	77.5	284.7	18.3
50	80.0	293.9	18.9
55	86.2	316.7	20.3
60	92.3	339.1	21.8

### Lesion production

**Fig 2:**
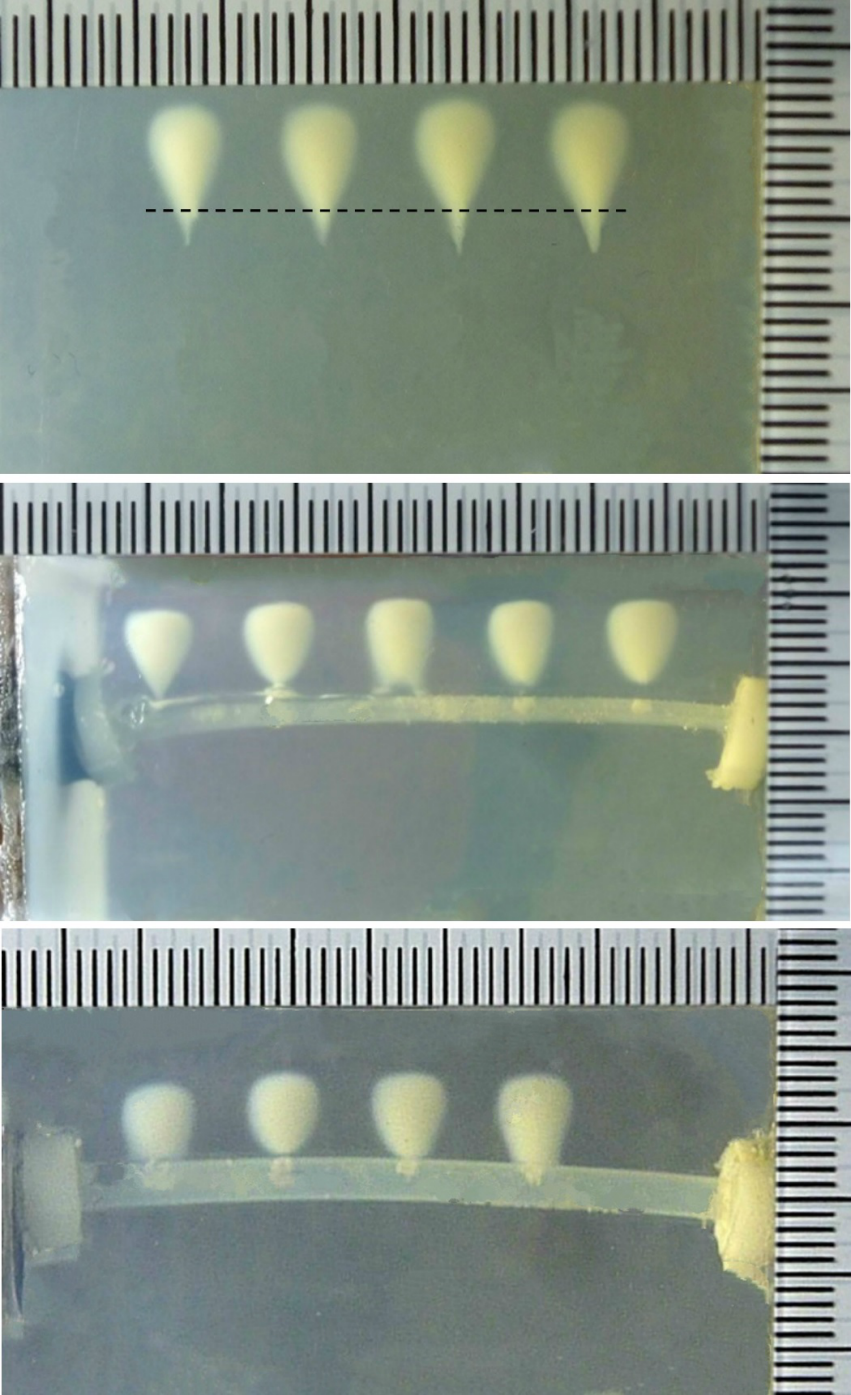
Representative photos of lesions produced (a) in a polyacrylamide gel phantom with 7% BSA after 100 s HIFU exposure at the peak negative pressure of -11.9 MPa, duty cycle of 20%, and pulse repetition frequency of 10 Hz (dashed line is the estimated HIFU focal plane), and close to (b) tubing 1, and (c) tubing 2 at the average flow speed of 267 m/s and 17 m/s, repectively.

Lesion produced in the BSA polyacrylamide phantom without the embedded tubing is shown in Fig 2A. It has the shape of a tadpole, gradually distorting from the symmetric lesion in the shape of a cigar to the asymmetric one with the much larger area extending towards the source, and the lesion size is 8.53±1.23×4.59±0.58 mm (length×width) at the PRF of 10 Hz and DC of 20%. The corresponding lesions when the HIFU focus was aligned to the middle of the tubing at the average flow speed of 267 m/s and 17 m/s in the tubing 1 and 2 are shown in Fig 2B and 2C, respectively. The lesion was only found in the prefocal region and close to the tubing, its size is 6.11±0.34×4.78±0.2 mm and 5.56±0.37×4.95±0.11 mm, respectively, decreasing the length by ~30% but increasing the width slightly by ~6%. One of the reasons for variations in the produced lesions is due to the curved tubing surface.

**Fig 3:**
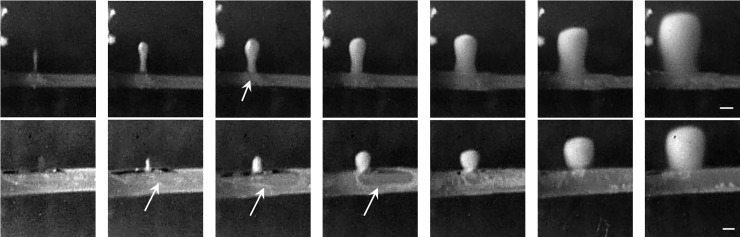
Representative images of lesion growth in a polyacrylamide gel phantom with 7% BSA after 100 s HIFU exposure at the peak negative pressure of -11.9 MPa, duty cycle of 20%, pulse repetition frequency of 10 Hz close to (a) tubing 1 and (b) tubing 2 with the circulating degassed water at the average flow speed of 267 m/s and 17 m/s, respectively. Arrows show bubbles accumulated inside the tubing during HIFU ablation. The scale bars present 2 mm.

Lesion growth with the ongoing of HIFU ablation is shown in Fig 3. Because of the large thickness of the tubing 1, significant acoustic reflection from the tubing wall occurred during HIFU ablation, and the lesion was produced about 1–2 mm prior to the anterior surface. Afterward, the asymmetric lesion was produced towards the source (Fig 3A). However, comparing with the tadpole-shaped lesion produced in the gel without the embedded tubing the sharp lesion tip disappeared and the tadpole tail seemed more cylindrical. Using the tubing 2, the initial lesion was more close to the tubing wall which may be due to the small wall thickness. In addition, some bubbles were found inside the tubing during HIFU ablation as shown by the arrows in Fig 3B and may be pushed forward by the acoustic radiation force by the HIFU burst from the induced lesion through the puncture site on the tubing wall.

**Fig 4:**
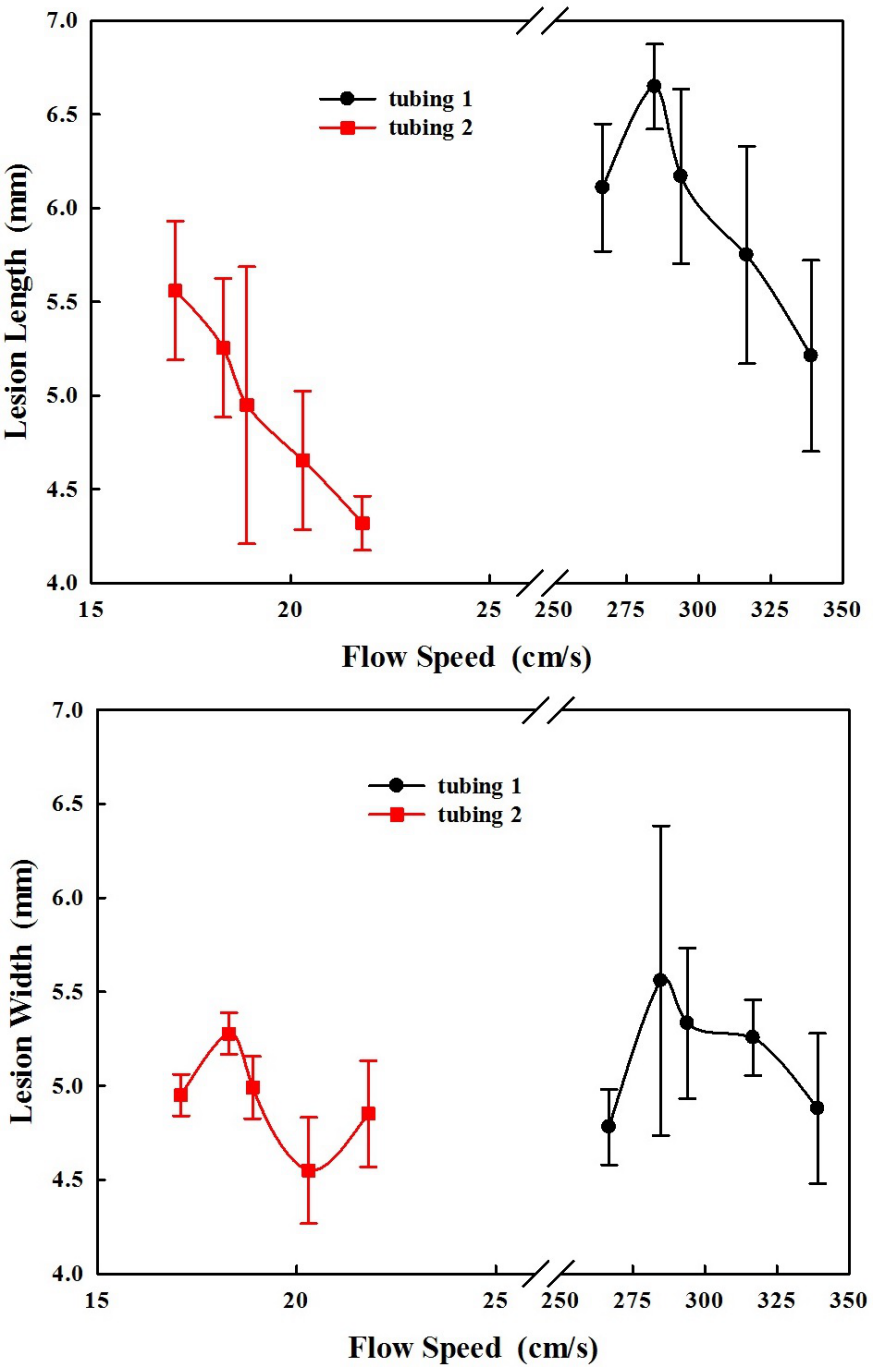
Dependence of the lesion (a) length and (b) width on the flow speed in these 2 tubing embedded in a polyacrylamide gel phantom with 7% BSA gel after 100 s HIFU exposure at the duty cycle of 20%, pulse repetition frequency of 10 Hz.

The dependences of lesion size (length and width) on the flow speeds in these 2 tubing are quite similar, lesion length decreasing with the flow speed while the influence on the lesion width is much less (Fig 4), despite the large variations in the experimental data which may be due to variations in the bubble nuclei concentration in the gel phantom and alignment of the HIFU focus to the tubing. The increase of the flow speed of 27% led to the slight decrease of lesion length by 17.4% and 22.4% in these 2 tubing, respectively.

### PCD signals

**Fig 5:**
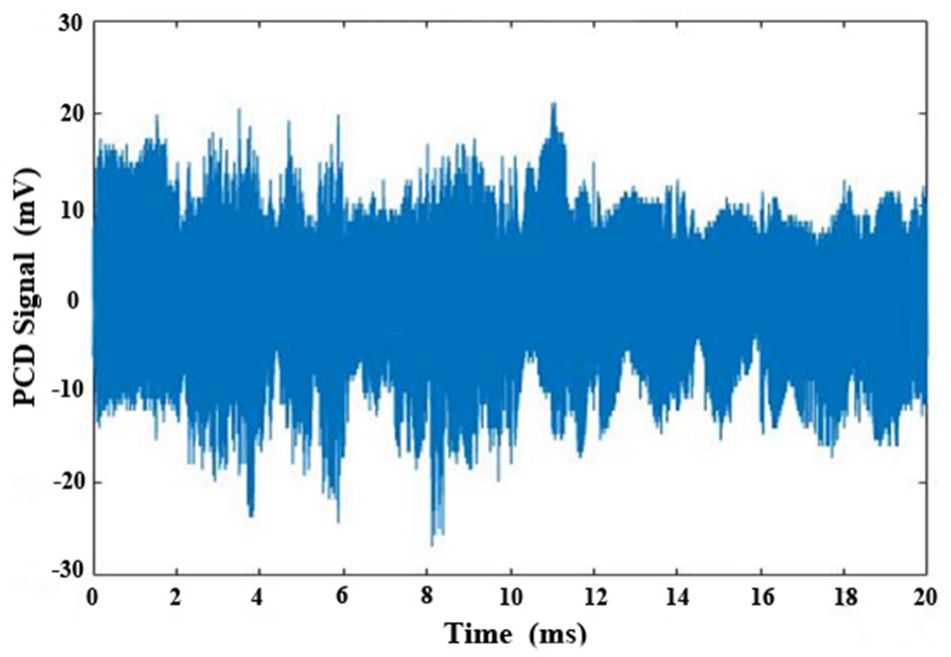
Representative passive cavitation detection (PCD) signals during a HIFU pulse at the pulse repetition frequency of 10 Hz and the duty cycle of 20%.

**Fig 6:**
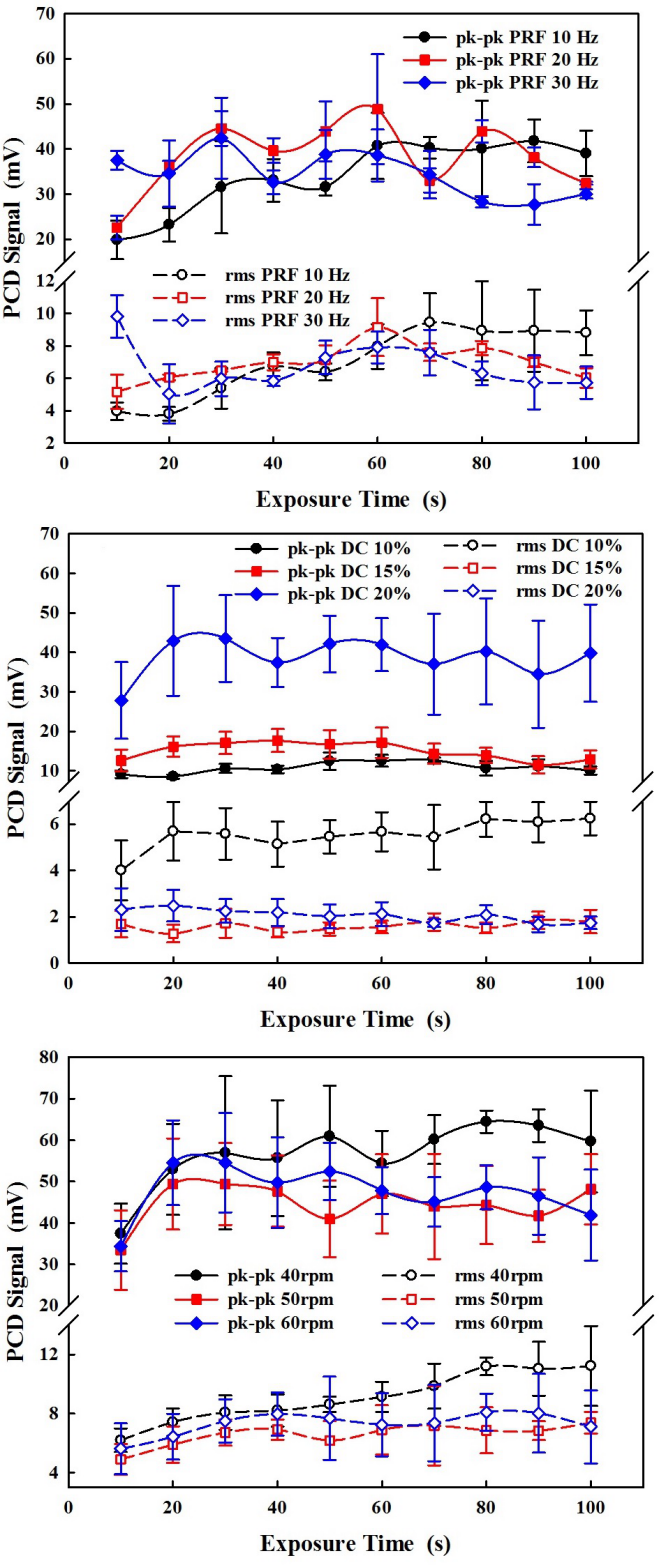
Variation of peak-to-peak and root mean square (rms) passive cavitation detection (PCD) signals during 10 s HIFU exposure from tubing 1 with the circulating degassed water (a) at varied pulse repetition frequency (PRF) of 10 Hz, 20 Hz, and 30 Hz, (b) at the varied duty cycle (DC) of 10%, 15%, and 20%, and (c) at the varied rotation speed of 40, 50, and 60 rpm. The control operation parameters are the PRF of 10 Hz, DC of 20%, and pump speed of 60 rpm.

The cavitation activities during HIFU ablation were measured by PCD, and the representative signal is shown in Fig 5. The effects of varied PRF (10–30 Hz), DC (10–20%), and average flow speed (267–339 m/s) on the PCD signals during HIFU ablation on the tubing 1 are shown in Fig 6. It is found that the varied PRFs have similar bubble cavitation, but high DC (e.g., 20%) and low flow speed (e.g., 267 m/s) may produce stronger bubble cavitation.

### Vessel rupture

**Fig 7:**
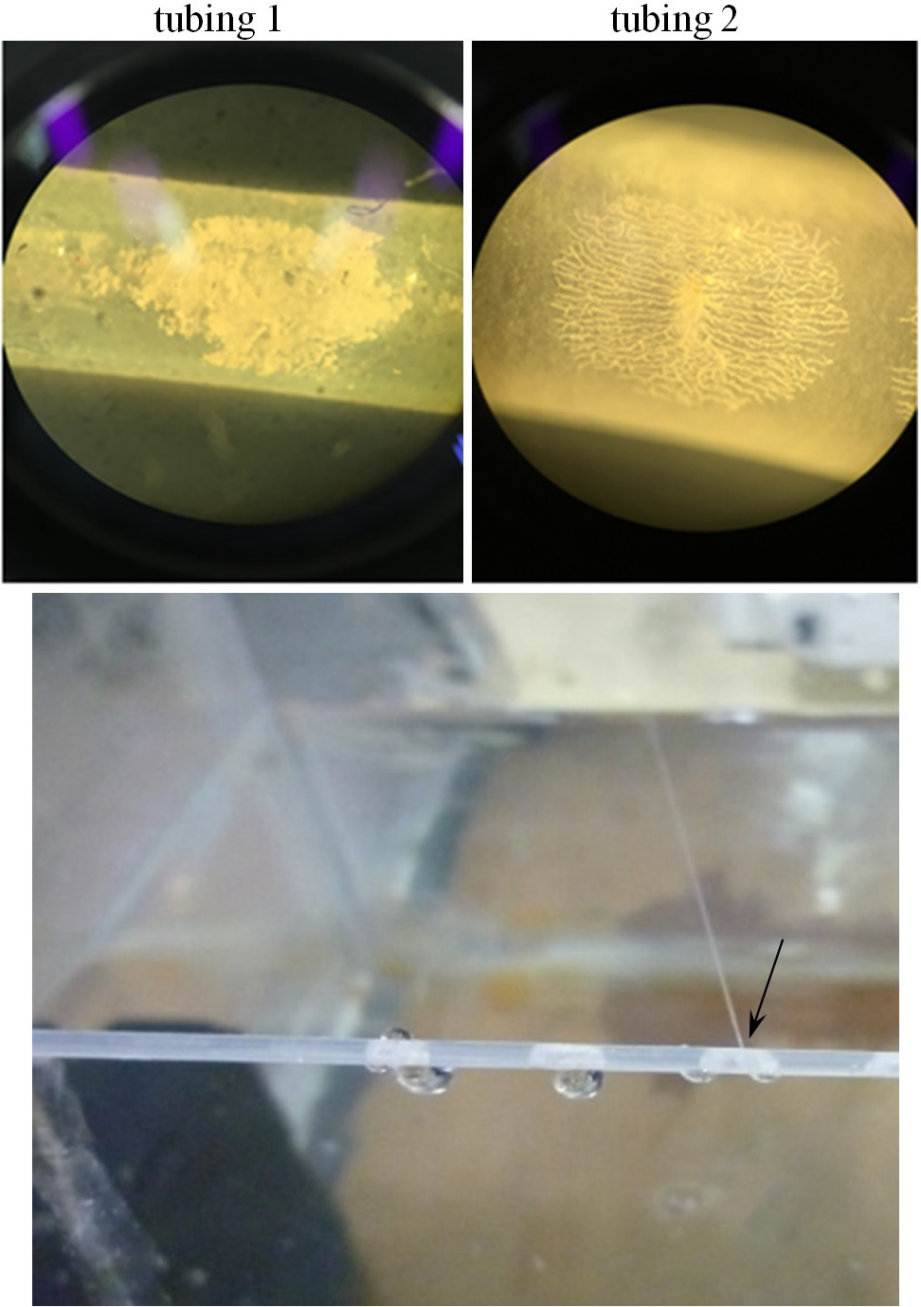
(a) Representative cracks found on these tubing after HIFU exposure under a light microscope, and (b) the water leakage from the puncture site.

**Fig 8:**
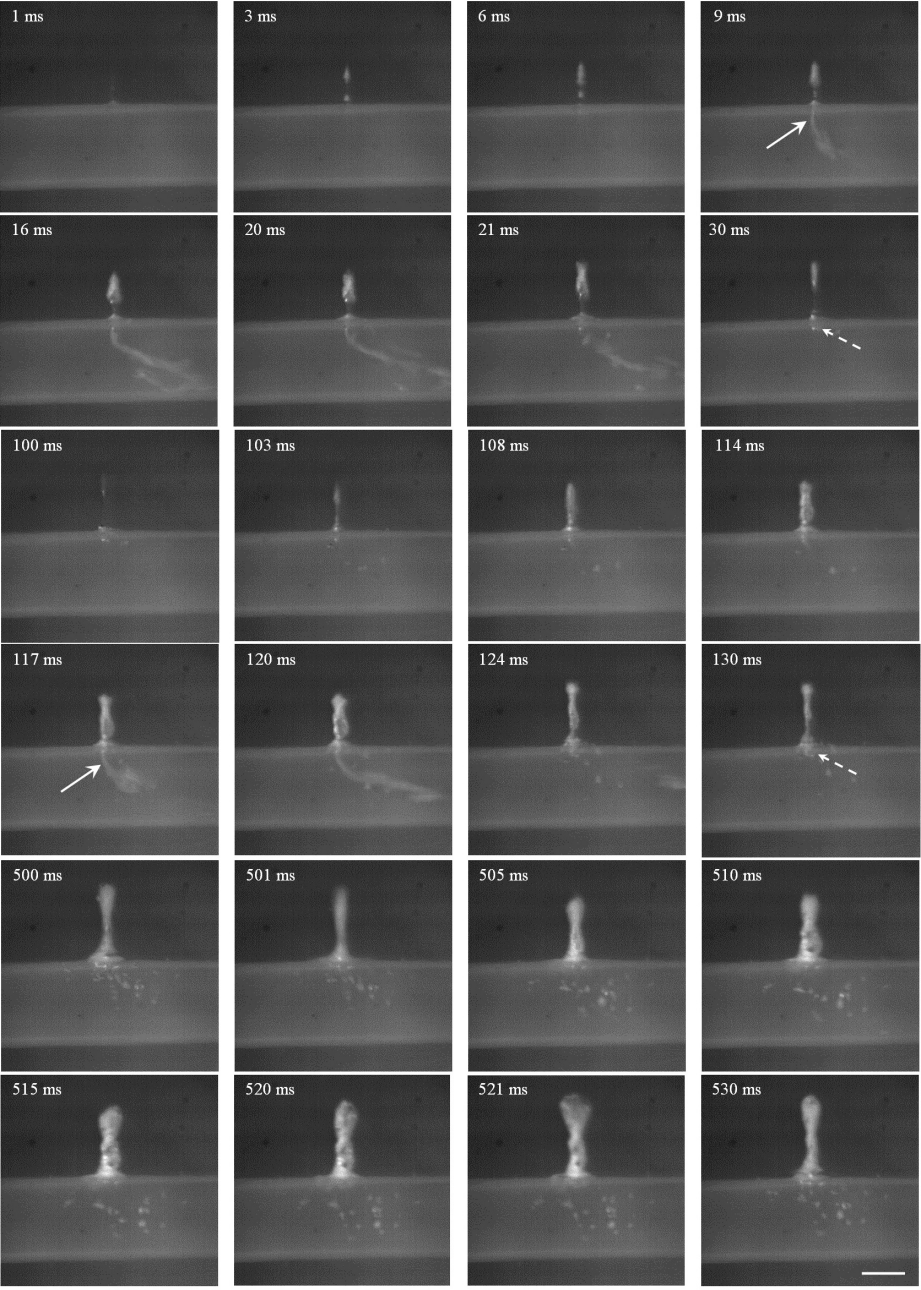
Representative high-speed photography of HIFU ablation with the focus aligned to the anterior surface of tubing 2 at the pulse repetition frequency of 10 Hz and duty cycle of 20%. Scale bar presents 2 mm, the arrow is the mist ejection toward the tubing, and the dashed arrow is the trapped bubble at the puncture site.

**Fig 9:**
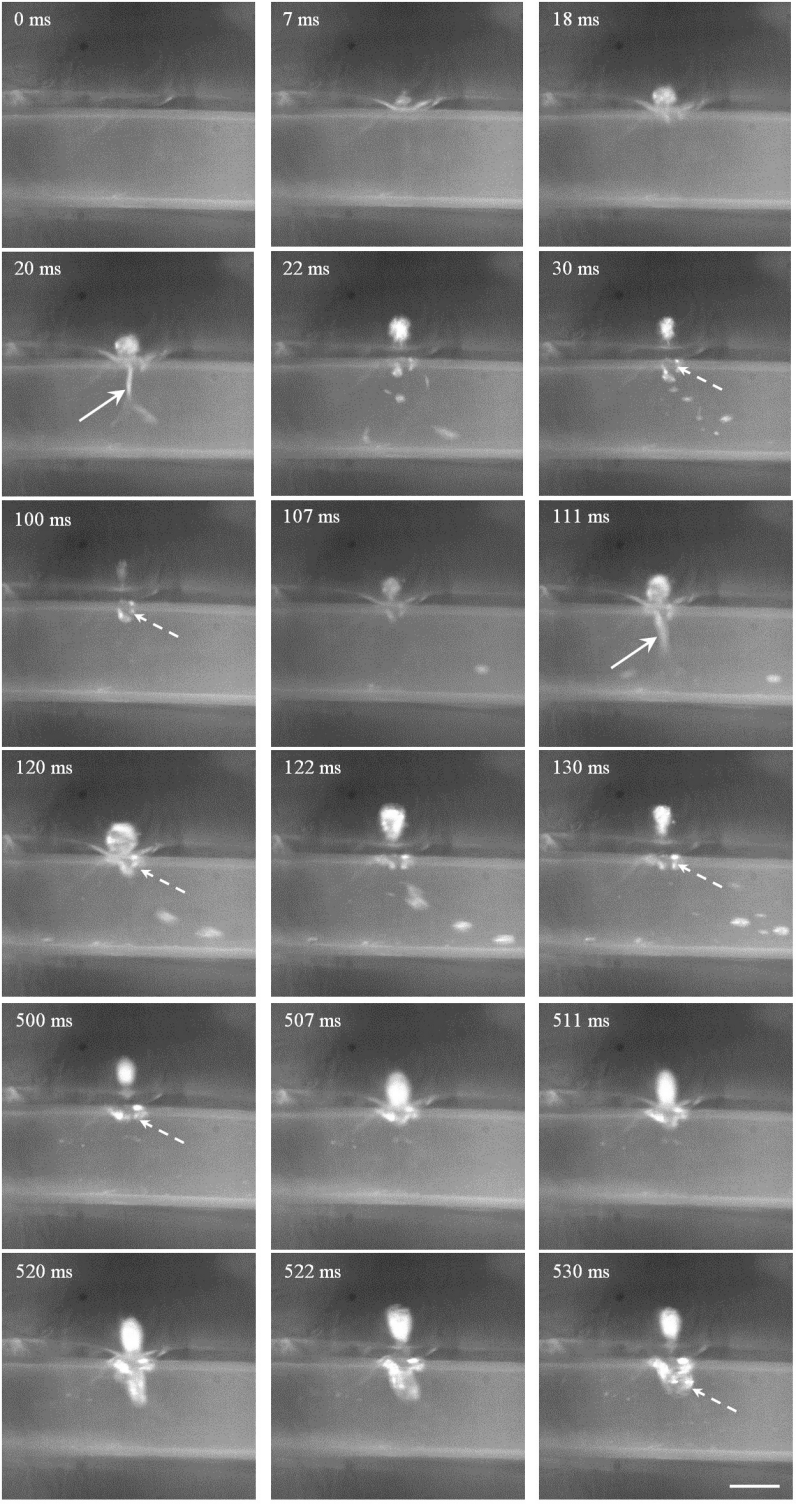
Representative high-speed photography of HIFU ablation with the focus aligned to the middle of tubing 2 at the pulse repetition frequency of 10 Hz and duty cycle of 20%. Scale bar presents 2 mm, the arrow is the mist ejection toward the tubing, and the dashed arrow is the trapped bubble at the puncture site.

**Fig 10:**
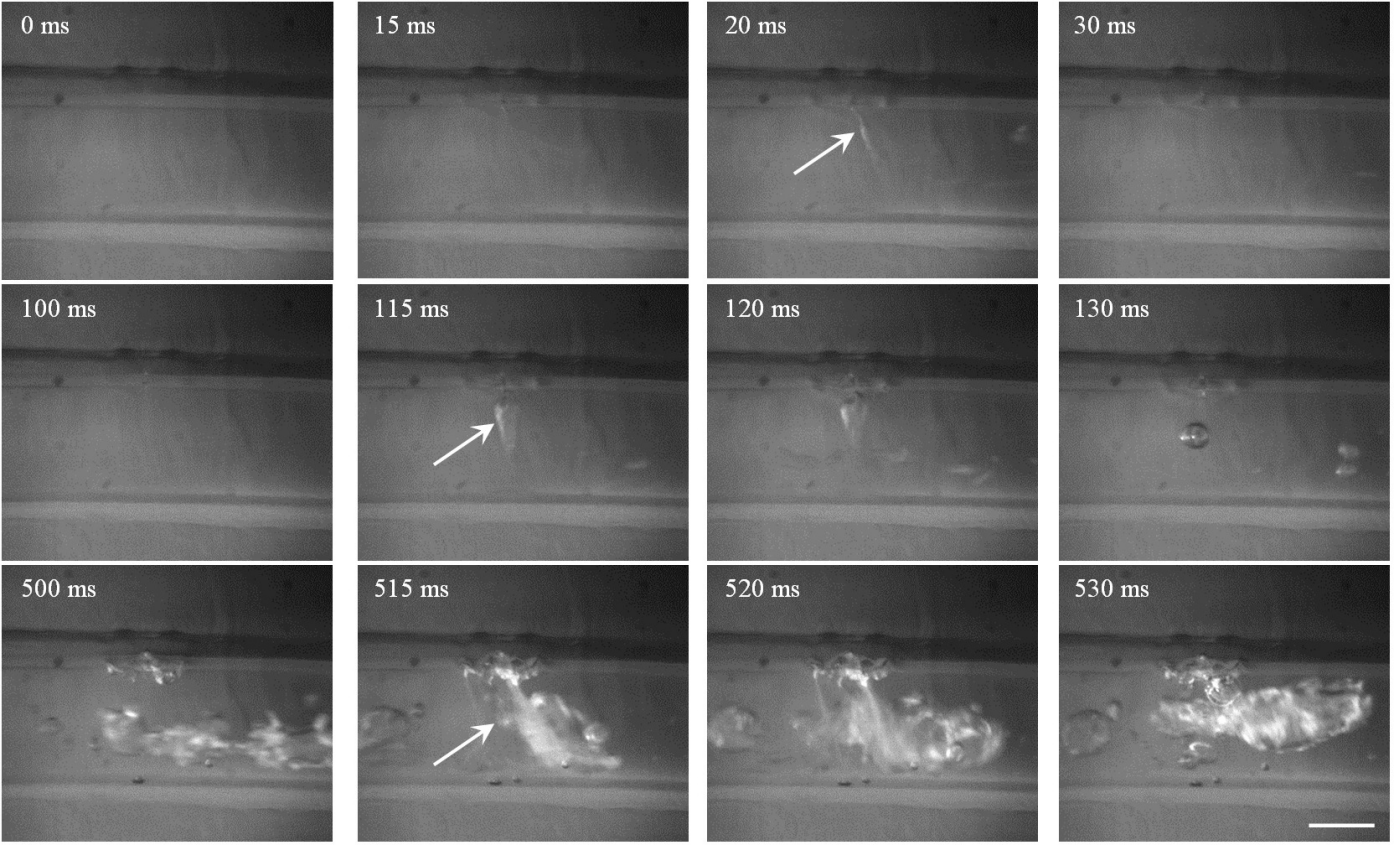
Representative high-speed photography of HIFU ablation with the focus aligned to the posterior surface of tubing 2 at the pulse repetition frequency of 10 Hz and duty cycle of 20%. Scale bar presents 2 mm, and the arrow is the mist/bubble ejection toward the tubing.

After HIFU ablation, pitting and cracks were found under a light microscope (CKX-41, Olympus, Shinjuku, Tokyo, Japan) on the anterior surface of these 2 tubing consistently at all testing conditions, and ejection and leakage of circulating water were found from the puncture sites (Fig 7). However, no ruptures were found on the posterior surface. In order to illustrate the mechanism of tubing rupture, high-speed photography was taken with different HIFU focus alignments (at the anterior surface, middle, and posterior surface of tubing). In all tested cases (*n* = 7 for each alignment), most of the tubing was ruptured by the first HIFU pulse within 20 ms (probability > 95%), and the characteristics of tubing rupture and bubble introduction are strongly dependent on the HIFU focus alignment (Figs 8–10).

When the HIFU focus was aligned to the anterior surface of the tubing, the bubble appeared at the focus immediately after starting HIFU exposure (e.g., 1 ms in Fig 8). With the ongoing of HIFU exposure, such bubble moved towards the transducer. At 9 ms, the tubing was ruptured, and bubble mist was ejected into the tubing through the puncture site. Immediately after the termination of HIFU exposure (e.g., 21 ms), the bubble in the gel phantom moved towards the transducer due to the hydrodynamic cavitation effect and then gradually shrank [18]. In addition, bubble trapped at the puncture site had a longer lifetime than that in the gel phantom because of the high surface tension for the bubble stability. Thus, it served as the acoustic scatterer for the incoming HIFU pulses and produced larger lesions in the gel phantom (*t* = 100–120 ms). However, no bubble ejection into the tubing was found after the 6^th^ HIFU pulse (*t* = 500–520 ms), which may be due to the blockage of puncture site by the denatured BSA protein at the high temperature and the gradually shifted location of bubble cavitation in the large head of the tadpole-shaped lesion.

Varying the HIFU focus alignment with respect to the tubing led to distinctive characteristics of lesion production and tubing rupture. When the HIFU focus was aligned to the middle of the tubing, a large bubble will be produced at the anterior surface of the tubing (e.g., *t* = 18 ms in Fig 9). The dynamics of such bubble and its interaction with the vessel wall may cause the vessel rupture and ejection of the bubble mist into the tubing after the 1^st^ HIFU pulse exposure (arrow at *t* = 20 ms). Immediately after the termination of HIFU exposure, the HIFU-induced bubble moved towards the transducer while some bubbles were trapped at the puncture site (dashed arrow at *t* = 30 ms). These trapped bubbles were sustainable during the interval of HIFU exposure and gradually became larger (*t* = 100 ms). At the 6^th^ HIFU pulse, the trapped bubble was about 1.5 mm in size (*t* = 530 ms). Meanwhile, the bubble mist ejection toward the intraluminal space was not found anymore, but bubbles were accumulated at the puncture site with a significant growth. In comparison, when the focus was aligned to the posterior surface of the tubing, no lesion was found after HIFU exposure of 1 s (Fig 10). Although the size of the initial rupture at the anterior surface was small (*t* = 20 ms), it enlarged quickly at the following exposure (*t* = 115 and 120 ms). At the 6^th^ HIFU pulse, the rupture was about 2.1 mm, and very large bubble (e.g., 6.2 mm in length) was produced and maintained inside the tubing (*t* = 500–530 ms). The produced large bubbles in the tubing did not dissolve for a quite long time (up to ten minutes in our experimental observation). The rupture at the anterior surface instead of the focused posterior surface may be due to the stronger cavitation in the prefocal region by scattering and reflection of HIFU-induced bubbles.

## Discussion

With the popularity of HIFU applications in clinics, more targets have been tried with satisfactory outcomes. Its safety to a vessel is a critical concern. In this study, the lesion formation and vessel disruption were evaluated in a tubing embedded gel phantom. The effects of tubing size, materials, and flow speed on lesion size were investigated. It is found that lesion length increased with the decrease of the flow speed while the dependence on lesion width was much less. HIFU exposure at high DC and the low flow speed produced the greater PCD signals, which may be mostly from the bubble activities in the tadpole lesion. Consistent tubing rupture was found at all HIFU settings. High-speed photography illustrated that the rupture was mostly produced within 20 ms and then bubbles penetrated into the tubing through the puncture site. At various alignments of the HIFU focus, different characteristics of lesion formation in the pre-focal region, the introduction of bubbles, and growth of puncture site were observed with the ongoing of HIFU exposure.

Vessel rupture was occasionally observed after clinical HIFU ablation. HIFU destroyed the shunt vessels, thereby enhancing the risk of angiorrhexis by the increased intravascular pressure [19]. The damaged vessels may play a critical role in the secondary tumor cell death, and then indirectly strengthen the destructive effect. Vessel rupture was mostly found adjacent to the target lesion and lying in the traveling path of the HIFU beams. Such a strategy of HIFU-induced tumor vessel damage may be promising. However, tumor rupture and detachment of cancer cells/emboli from the primary site may also be involved in the formation of metastasis or embolism [20]. In addition, the introduction of a large bubble in the vessel should be avoided. Pulmonary (venous) and cerebral arterial air embolism is life-threatening and may cause immediate cardiovascular collapse as shown during Nd:YAG laser-induced hyperthermia for the lung cancer and malignant liver tumors [21,22]. Therefore, investigating the mechanisms of HIFU-induced vessel rupture would help the further development of HIFU technology, either reducing the unintended injury or increasing the beneficial effect.

The mechanism of HIFU-induced vessel rupture is not fully understood. Excessive temperature elevation (e.g., 100°C) was considered the main factor of the vessel hemorrhage [23]. But large boiling bubble was not found here in the tested tubing using the photography. Rupture rate of rabbit femoral vessels after HIFU exposure (peak pressures of 4–17 MPa, exposure of 10, 60, and 180 s) at the frequency of 0.68 MHz was found higher than that at 2.02 MHz [24]. The focus of the 0.68 MHz beam encompassed the vessels and surrounding tissues, whereas that of the 2.02 MHz beam aimed only the target vessel and the main damage mechanism is the thermal coagulation. Both the arteries and veins were constricted or occluded often without the vessel rupture due to the induced spasms at the low frequency. The stiffening of the coagulated surrounding tissue produced at the high frequency may prevent the vessel rupture, which is similar to our observation (Fig 8). In our high-speed photography, vessel phantom constriction was not found, and tubing puncture occurred before the lesion formation on the tubing wall. In the lithotripter shock wave-induced renal injury, shear stress and cavitation are considered two competing mechanisms. Shear deformation due to a shock front propagating through a heterogeneous medium could be accumulated if the relaxation time of the vessel is comparable to the PRF of delivered pulses [25]. However, intraluminal bubble expansion and collapse were not found here. Thus, the tubing rupture may be due to the shear stress by the bubble cavitation. Such a hypothesis needs further confirmation using *ex vivo* vessel tissues. In addition, it is noted that HIFU ablation is not a standardized procedure, and its intensity and insonation are usually modified throughput a treatment [26]. Pre-damage may be induced under certain insonation mode, and then the vessel may be destructed by the following insonation if the intensity is beyond a critical threshold.

Bubble activities and the occurrence of tissue vaporization (or the formation of the boiling bubble) during HIFU exposure could be monitored in real time by PCD. Stable and inertial cavitation signified by subharmonic and ultraharmonics emission and by broadband acoustic emissions associated with bubble collapse, respectively; whereas a low-frequency emission was associated with the tissue or fluid vaporization and boiling [27]. HIFU treatments of porcine femoral arteries immersed in the degassed 0.9% PBS showed that subharmonic emission was predictive of vessel rupture which is figured out as a 3% decrease in the flow rate [27]. Using PCD signals as a real-time feedback, acoustic cavitation could be reduced by adjusting the HIFU power. As a result, the potential vessel rupture or clinical hemorrhage may be avoided without compromising HIFU ablation capability. The relationship between vessel rupture inside the tissue or gel phantom and spectrogram of PCD signals, especially the tiny rupture by the first HIFU burst, is required for the development of such a control algorithm.

The influence of blood vessels on the temperature distribution during HIFU ablation has been simulated in a 3D acoustics-thermal-fluid coupling model using linear/nonlinear Westervelt, BioHeat, and the nonlinear Navier–Stokes equations [28–32]. Both the convective cooling and acoustic streaming can significantly change the temperature field and thermal lesion near the blood vessels (within a beam width). Subsequently, the significant reduction in lesion volume (about 40% for a parallel orientation and 20% for a perpendicular case) was found [29]. Such an influence became insignificant for gaps larger than a beam width. Although the acoustic intensity in the simulation could be beyond the threshold for cavitation, boiling, and highly nonlinear propagation, some factors involved in the vessel rupture, such as the bubble cavitation, the production of the liquid jet, and the interaction with the vessel wall, are not included. Great efforts are required to establish a more complicated but realistic model, and then the simulation results could be compared with experimental observation to further understand the mechanisms.

There are several limitations of this study. Firstly, the tubing has different properties from the vessel despite in the similar size. The Young’s modulus of silicone rubber and PVC soft are 1–50 MPa and 20–50 MPa, respectively, which is higher than the reported values of arteries and vessels, 0.04–1.6 MPa [33]. The conclusion made here may not be extrapolated to the *in vivo* tissue directly, but provide some preliminary insights. Whether similar characteristics of bubble dynamics, lesion production, and vessel rupture can be found in the *ex vivo* vessels needs further investigation. Secondly, the dependence of the acoustic intensity, peak pressure, and beam size on the tubing rupture at varied settings using a higher number of samples may illustrate the dominate parameter and determine a threshold as the guidelines for the HIFU applications. Thirdly, the role of excessive heating on the vessel rupture was unknown in the current setup. Higher acoustic power is required to produce a large boiling bubble in a short exposure, and appropriate thermometry (e.g., thermocouple) could monitor the temperature elevation and then may associate with the onset of vessel rupture. Finally, more conditions, such as intraluminal pressure regulation, induction of flow infusion, and the presence or absence of intraluminal microbubbles, should be included to simulate the *in vivo* environment [27,34]. A microscope could monitor the leakage of intraluminal fluorescence marker while a fiber-optic hydrophone sensor mounted on the vessel chamber could allow the focus alignment within 150 μm and monitor the acoustic cavitation emissions during the ultrasonic exposures. However, because of the optical opaqueness of the tissue, the direct observation of bubble interaction with the vessel is still challenging.

## Conclusions

In summary, HIFU ablation to the tubing aligned at the focus perpendicularly can also produce the lesion in the gel phantom. Lesion size and shape depend on the tubing material, geometry, and the flow speed. Meanwhile, consistent tubing rupture was also found after the exposure. High-speed photography illustrated that puncture of tubing occurred by the first HIFU burst within 20 ms and the subsequent lesion formation, bubble introduction, and growth of the puncture site were highly dependent on the initial HIFU focus alignment with respect to the tubing. This investigation illustrated some mechanisms of HIFU-induced vessel rupture, from which the technical improvement of either enhancing or reducing such phenomenon (e.g., HIFU excitation modalities, energy delivery strategy, treatment monitoring, and feedback control) may be developed to widen the HIFU applications.
